# Readiness and willingness of Malaysian community pharmacists in providing vaccination services

**DOI:** 10.1186/s40545-022-00478-0

**Published:** 2022-11-12

**Authors:** Wei Chern Ang, Mohamad Syafuan Fadzil, Fatin Najihah Ishak, Nassrah Norissya Adenan, Mohamad Haniki Nik Mohamed

**Affiliations:** 1 Clinical Research Centre, Ministry of Health Malaysia, Hospital Tuanku Fauziah, Kangar, Perlis Malaysia; 2 Department of Pharmacy, Hospital Tuanku Fauziah, Ministry of Health Malaysia, Kangar, Perlis Malaysia; 3 Immunisation Advocacy Chapter, Malaysia Pharmacist Society, Puchong, Selangor Malaysia; 4 Kulliyyah of Pharmacy, International Islamic University Malaysia, Kuantan, Pahang Malaysia

**Keywords:** Community pharmacists, Readiness, Willingness, Vaccination services, Malaysia

## Abstract

**Background:**

Vaccination is an effective public health intervention in reducing morbidity and mortality of infectious diseases. Compared to other countries where community pharmacists (CPs) administer vaccines, CPs in Malaysia are not authorised. This study aimed to assess CPs' readiness and willingness to provide vaccination in Malaysia, identify potential barriers to and factors supporting the provision of this service.

**Methods:**

A cross-sectional study was conducted among Malaysian CPs from April to June 2021. A validated online questionnaire was distributed through social media, instant messaging, email, and pharmacy societies.

**Results:**

Of 492 CPs recruited throughout Malaysia, 439 (89.2%) expressed willingness to provide vaccination services to the public, 403 (81.9%) agreed with the accessibility of community pharmacies to the public, and 73.4% agreed that their role in vaccination could help to improve the overall vaccination coverage rate. The lack of pharmacist training in vaccination and concerns on maintaining patient safety were identified as barriers to CPs' implementation of vaccination services, with 52.8% and 47.8% of them agreeing, respectively. Training sessions and operational guidelines on providing vaccination services are required to overcome the barriers.

**Conclusion:**

CPs in Malaysia were ready and willing to provide vaccination services to the public. However, the implementation demands training workshops and re-evaluation of CPs in public vaccination programmes by Malaysian healthcare policymakers.

## Background

Poor vaccination coverage continues to be a significant public health concern [1]. In Malaysia, vaccines have been distributed since the early 1950s through the National Vaccination Programme to protect Malaysian residents from vaccine-preventable diseases [2]. In addition to personal protection, vaccination can help the entire community by reducing the spread of infectious diseases when most of the population is vaccinated. Mass vaccination can also protect those who cannot be vaccinated for health reasons, such as allergies or immunocompromised.

In Malaysia, vaccines have been administered for the management of the COVID-19 pandemic since February 2020. As of 29th March 2022, 79.0% of the population have received two COVID-19 vaccine doses, while 48.1% received their booster dose [3]. The majority of the vaccinators are healthcare providers from the government sector, but private healthcare professionals including physicians and nurses, are also actively involved. Nevertheless, pharmacists were not authorised to administer vaccine.

Community pharmacists (CPs) play a vital role in contributing to public health through accessibility, distribution and vaccination advocacy [4]. Many countries have permitted CPs to provide vaccination services to increase the vaccination rate, including Norway, Greece, Portugal, the United Kingdom, and Estonia [5].

A cross-sectional survey from February to April 2016 in Riyadh, Saudi Arabia [5] revealed that most CPs were willing to provide vaccination services although they were not authorised to vaccinate. However, overcoming the main barriers, such as the lack of training and concerns on maintaining patient safety, are the keys to successfully implementing such services. A later study conducted in Poland between February and August 2020 during the initial phase of the COVID-19 pandemic [4] revealed that the CPs trained in vaccination improved the overall vaccination rate and played an essential role in advertising and promoting vaccination. Meanwhile, CPs who were not trained expressed their opinion that pharmacies were not adapted to vaccination and there were not enough training courses for pharmacists [4].

Involving CPs in administering vaccination is beneficial as community pharmacies are open for extended hours, in strategic locations including rural areas, which demonstrated higher accessibility compared with the other healthcare facilities [4, 5, 6, 7] while reducing the workload of the healthcare system [1]. Nevertheless, CPs' readiness and willingness to provide vaccination services in Malaysia have not been explored. Recently, the Ministry of Health Malaysia (MOHM) has considered an expansion and authorisation in the role of pharmacists in vaccination [8]. However, it is unclear whether this service will be implemented. This new idea of extended pharmacy service could be fundamental during this COVID-19 pandemic as pharmacists can significantly impact the vaccination coverage rate for the Malaysian population and actively combat infectious diseases with other healthcare professionals. Therefore, the objective of this study is to assess the readiness and willingness of CPs to provide vaccination services and identify barriers to and factors for implementing such services.

## Methods

A cross-sectional study was conducted among pharmacists working in community pharmacies in Malaysia. The research instrument was based on the validated English questionnaire from the previous research conducted in Poland [4]. The questionnaire was content validated by academic experts, public health specialists and pharmacists to adapt to the Malaysian healthcare system. The questionnaire further underwent face validation by three CPs excluded from the actual study. The finalised self-administered questionnaire using Google Form (Google Inc., Mountain View, CA) in the English language was distributed via social media, instant messaging platform, email, Malaysian Pharmacists Society (MPS) and Malaysian Community Pharmacy Guild (MCPG). Informed consent was obtained prior to answering the questionnaire. Provisionally registered pharmacists (PRPs) working in community pharmacies were excluded from this study.

The questionnaire is divided into five main sections: sociodemographics of the CPs and the pharmacies, readiness to provide vaccination services in pharmacies (4 items), barriers to vaccination (9 items), factors necessary to implement vaccination services in pharmacies (11 items) and five closing questions on willingness and opinions. Each questionnaire item was assessed using a 4-point Likert scale (1: strongly disagree; 4: strongly agree) except for the sociodemographics and open questions.

The sample size required was 340 respondents, calculated by the population proportion formulae [9] using 50% sample proportion to obtain the maximum sample size, and 2889 community pharmacies registered in Malaysia [10] with a 5% margin of error and 95% confidence level.

All statistical tests were performed using SPSS Version 20.0 (IBM Corp., Armonk, NY). Continuous variables were presented as means and standard deviation (SD) and categorical variables as frequencies and percentages. Logistic regressions were conducted to assess the factors on vaccination willingness and barriers affecting pharmacists' readiness for vaccination. The statistical significance was set at *p* < 0.05.

## Results

A total of 492 respondents with a mean age of 32 years participated. They were primarily female, held a bachelor's degree as the highest education level, worked in chain pharmacies and urban areas (Table 1). There were only 56 (11.4%) pharmacists who had received vaccination training**.**

**Table 1:** Characteristics of respondents and pharmacies (*n* = 492)

Characteristics	*n* (%)/mean ± SD
Age (year old)	32 ± 11.0
< 30	192 (39.0)
30–40	198 (40.3)
> 40	102 (20.7)
Working experience in community pharmacy (year)	5 ± 9.0
< 5	270 (54.9)
5–10	93 (18.9)
> 10	129 (26.2)
Gender	
Male	173 (35.2)
Female	319 (64.8)
Highest education	
Bachelor’s degree	427 (86.8)
Master	62 (12.6)
PhD	3 (0.6)
Position	
Owner	142 (28.9)
Staff	350 (71.1)
Received vaccination training	
Yes	56 (11.4)
No	436 (88.6)
Types of pharmacy	
Chain	265 (53.9)
Independent	227 (46.1)
Pharmacy location	
Urban	395 (80.3)
Rural	97 (19.7)

**Fig. 1:**
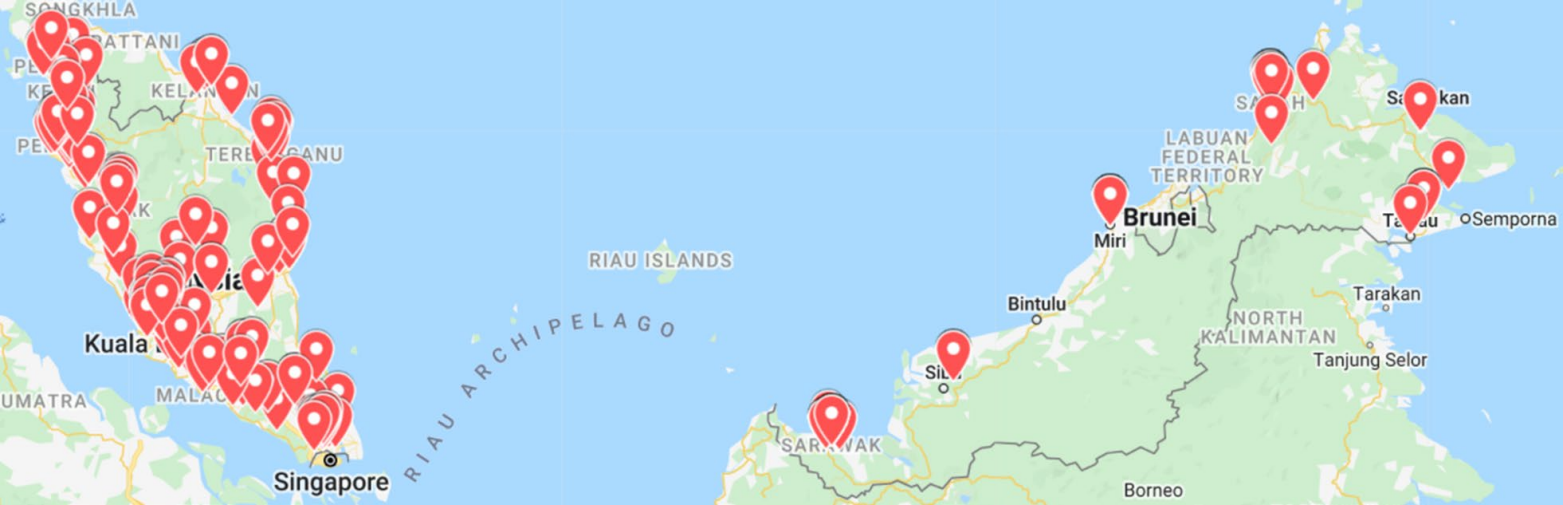
The distribution of respondents in Malaysia (*n* = 492) based on postcode. Source: Google Maps (Google Inc., Mountain View, CA)

As shown in Fig. 1, the CPs sampled were nationally representative of the CPs' and population distribution in Malaysia, except for the coastal town of Bintulu in which no response was received.

On the readiness indicators, 179 (36.4%) and 264 (53.7%) participants strongly agreed and agreed that CPs have a good understanding of vaccines and their indications, respectively (Table 2). Most pharmacists (81.9%) strongly advocated that community pharmacies are easily accessible to the community and 68.7% strongly believed this can increase the vaccination rate in certain groups, such as the elderly. The majority (72.6%) also strongly agreed that pharmacists could play an essential role in public vaccine advertising and promotion**.**

**Table 2:** Readiness of pharmacists to provide vaccination services in community pharmacies

Issue	Level of agreement			
	Strongly agree, *n* (%)	Agree, *n* (%)	Disagree, *n* (%)	Strongly disagree, *n* (%)
Community pharmacists have good knowledge of vaccine and their indications	179 (36.4)	264 (53.7)	47 (9.6)	2 (0.4)
Community pharmacists are easily accessible to the community	403 (81.9)	87 (17.7)	2 (0.4)	0 (0)
Providing vaccination through community pharmacy will improve the overall vaccination coverage rate	361 (73.4)	122 (24.8)	9 (1.8)	0 (0)
Providing vaccination through community pharmacy will improve the vaccination coverage in a certain group of patients, such as adult and elderly	338 (68.7)	144 (29.3)	9 (1.8)	1 (0.2)
The vaccination programme administered in community pharmacies is cost-effective	240 (48.8)	199 (40.4)	46 (9.3)	7 (1.4)
Community pharmacists can play an important role in advertising and promoting vaccination	357 (72.6)	131 (26.6)	4 (0.8)	0 (0)

On the barriers to providing vaccination services (Table 3), almost all (52.8% strongly agreed; 40.4% agreed) concurred on the lack of pharmacists training to deliver vaccination and believed that patient safety might be compromised (47.8% strongly agreed; 45.9% agreed). Most of the participants also expressed their concerns about handling vaccines, storage, and disposal of sharp items as barriers to the vaccination services, with 127 (25.8%) strongly agreeing and 197 (40.0%) agreeing with the statement. Regarding the implementation of vaccination services by CPs that may cause conflict with other healthcare professionals who are eligible to vaccinate, the opinion was almost equally divided, with 16.5% strongly agreeing and 35.6% agreeing. In comparison, 16.7% strongly disagreed and 31.3% disagreed.

**Table 3:** Barriers that influence pharmacists' readiness to provide vaccination

Issue	Level of agreement			
	Strongly agree, *n* (%)	Agree, *n* (%)	Disagree, *n* (%)	Strongly disagree, *n* (%)
Pharmacists are busy and have no time to provide vaccination	28 (5.7)	131 (26.6)	243 (49.4)	90 (18.3)
Vaccination will add more work to pharmacists	48 (9.8)	245 (49.8)	136 (27.6)	63 (12.8)
Patient safety is a concern	235 (47.8)	226 (45.9)	24 (4.9)	7 (1.4)
Lack of pharmacists training in vaccination	260 (52.8)	199 (40.4)	27 (5.5)	6 (1.2)
Pharmacists are less trusted by patients to provide vaccination services	38 (7.7)	123 (25.0)	243 (49.4)	88 (17.9)
Pharmacy is not equipped to provide vaccination service	101 (20.5)	218 (44.3)	123 (25.0)	50 (10.1)
Conflicts with other professionals who are eligible to vaccinate	81 (16.5)	175 (35.6)	154 (31.3)	82 (16.7)
Concerns about handling vaccines, storage, and disposal of sharps item	127 (25.8)	197 (40.0)	112 (22.8)	56 (11.3)
Pharmacists are not comfortable using needles	41 (8.3)	120 (24.4)	225 (45.7)	106 (21.6)

Among the important factors for the implementation of pharmacy vaccination services (Table 4), most pharmacists (68.9%) strongly agreed that the certification of pharmacists in providing vaccination is an essential element, followed by cooperation between pharmacists and health professionals (61.4%) and continuous education and training workshops (61.2%). In addition, 263 (53.5%) of participants agreed that adequate reimbursement or remuneration of pharmacies is an essential factor in their ability to provide vaccination services, followed by 245 (49.8%) who agreed with providing specific space for vaccination and 271 (55.1%) who agreed with providing specific space to store vaccines.

**Table 4:** Important factors for the implementation of pharmacy vaccination services

Issue	Level of agreement			
	Strongly agree, *n* (%)	Agree, *n* (%)	Disagree, *n* (%)	Strongly disagree, *n* (%)
More university education and training courses on vaccination administration for pharmacists	271 (55.1)	202 (41.1)	17 (3.5)	2 (0.4)
Continuous education and training workshops on vaccination	301 (61.2)	182 (37.0)	7 (1.4)	2 (0.4)
Adequate reimbursement or remuneration	263 (53.5)	210 (42.7)	17 (3.5)	2 (0.4)
Patients’ acceptance on the implementation of vaccination administered by pharmacists	220 (44.7)	259 (52.6)	11 (2.2)	2 (0.4)
Providing a specific space for vaccination in the pharmacies	245 (49.8)	229 (46.5)	16 (3.3)	2 (0.4)
Providing a specific space to store vaccine	271 (55.1)	208 (42.3)	12 (2.4)	1 (0.2)
More pharmacists and staff in pharmacies to allocate time and provide an individual approach to patients for vaccination services	224 (45.5)	247 (50.2)	19 (3.9)	2 (0.4)
Reduce the workload of technical tasks for pharmacists (e.g., entering invoices, verifying supplies) to allocate more time for vaccination services	185 (37.6)	241 (49.0)	62 (12.6)	3 (0.6)
Cooperation between pharmacists and health professionals	302 (61.4)	187 (38.0)	2 (0.4)	1 (0.2)
Support of medical and nursing associations	271 (55.1)	216 (43.9)	4 (0.8)	1 (0.2)
Certification of pharmacists in providing vaccination services	339 (68.9)	146 (29.7)	5 (1.0)	2 (0.4)

Regarding the readiness and barriers to providing vaccination services, almost all (99.2%) CPs expressed readiness, and 65.2% agreed that they might be experiencing some barriers in administering vaccines (Table 5).

**Table 5:** Mean score and level of readiness, barriers and essential factors to implement vaccination services in community pharmacies

Domain	Score, mean ± SD	Level, *n* (%)	
		Low	High
Readiness (score of 24)	21.3 ± 2.86	4 (0.8)	488 (99.2)
Barrier (score of 36)	20.4 ± 6.98	171 (34.8)	321 (65.2)
Factor (score of 42)	34.6 ± 4.49	3 (0.6)	489 (99.4)

Despite an almost absolute (99.2%) readiness, only 439 (89.2%) expressed willingness to administer vaccines at their current pharmacies. Hence, logistic regression was not performed on readiness but on willingness to administer vaccination (Table 6). Only the age of pharmacists and the location of pharmacies independently affect willingness. The p-value for the Hosmer–Lemeshow goodness-of-fit test is 0.115 (*p* > 0.05) means the model fits well. There was an additional factor of pharmacists' position in the final model: an increase in the age of pharmacists, being a pharmacist staff and working in rural areas improves willingness.

**Table 6:** The impact of characteristics of pharmacists and pharmacies on willingness to provide vaccine services

Characteristics	cOR (95% CI)	*p*-value	aOR (95% CI)	*p*-value
Age (year old)	1.03 (1.00, 1.07)	0.027	1.08 (1.04, 1.12)	< 0.001
Working experience in community pharmacy (year)	1.03 (0.99, 1.06)	0.129		
Gender		0.619		
Male	1.00 (ref.)			
Female	1.17 (0.64, 2.15)			
Highest education		0.056		
Bachelor's degree	1.00 (ref.)			
Master	0.25 (0.06, 1.04)			
PhD	n/a			
Position		0.292		0.004
Owner	1.00 (ref.)		1.00 (ref.)	
Staff	1.44 (0.73, 2.82)		3.58 (1.52, 8.46)	
Received vaccination training		0.083		
Yes	1.00 (ref.)			
No	3.58 (0.85, 15.12)			
Types of pharmacy		0.196		
Chain	1.00 (ref.)			
Independent	0.68 (0.38, 1.22)			
Pharmacy location		0.001		< 0.001
Urban	1.00 (ref.)		1.00 (ref.)	
Rural	2.85 (1.55, 5.23)		3.55 (1.88, 6.70)	

The model on barriers fits well, with a non-significance (*p* = 0.312) in the Hosmer–Lemeshow test. In both simple and multiple logistic regressions, pharmacists working as staff, in rural areas and have yet to receive vaccination training perceived more barriers to providing vaccination services (Table 7).

**Table 7:** The impact of characteristics of pharmacists and pharmacies on barriers to providing vaccination services

Characteristics	cOR (95% CI)	*p*-value	aOR (95% CI)	*p*-value
Age (year old)	1.00 (0.98, 1.02)	0.515		
Working experience in community pharmacy (year)	0.99 (0.97, 1.03)	0.051		
Gender				
Male	1.00 (ref.)			
Female	1.47 (1.00, 2.16)			
Highest education		0.159		
Bachelor's degree	1.00 (ref.)			
Master	0.65 (0.38, 1.11)			
PhD	0.25 (0.02, 2.77)			
Position		0.005		0.001
Owner	1.00 (ref.)		1.00 (ref.)	
Staff	1.78 (1.20, 2.67)		1.96 (1.29, 2.98)	
Received vaccination training		< 0.001		0.003
Yes	1.00 (ref.)		1.00 (ref.)	
No	3.10 (1.75, 5.47)		3.02 (1.68, 5.42)	
Types of pharmacy		0.084		
Chain	1.00 (ref.)			
Independent	0.72 (0.50, 1.05)			
Pharmacy location		0.001		< 0.001
Urban	1.00 (ref.)		1.00 (ref.)	
Rural	2.57 (1.50, 4.41)		2.57 (1.48, 4.45)	

In the closing questions, most were willing to provide services to as many patients as possible per day without service fees after receiving free training and vaccine from the government as part of social responsibility in combating the COVID-19 pandemic. Their median (± interquartile range) willingness to pay to enrol in vaccination certification training was MYR100 ± 250 (≈US$23.64 ± 59.29). The respondents suggested that theoretical and practical training in storage, administration technique, and handling of an emergency, such as anaphylactic reaction, should be included in the vaccination training. Most of them also prefer to have at least 1 or 2 days of training to have good skills in vaccine administration. The most common types of vaccination they would like to administer are influenza, followed by COVID-19 vaccines, typhoid and hepatitis vaccines.

## Discussion

To the best of our knowledge, this is the first study in Malaysia to assess CPs' willingness to provide a new extended pharmacy service on vaccine administration. Our findings show that most Malaysian CPs involved in this study were willing to administer vaccines. According to the US Centers for Disease Control and Prevention (CDC), vaccines are still the best public health tool for protecting people from COVID-19, which can cause delayed transmission and lower the chances of new variants arising: children 5 years and older should get fully vaccinated against COVID-19, whilst people over the age of 18 years should obtain a booster shot [11], which are widely supported worldwide. The continuous emergence of the COVID-19 variants may require additional support from underutilised healthcare professionals such as CPs to increase the vaccination coverage rate. This study shows their readiness and willingness to administer vaccines. The CPs' willingness to provide vaccination services was higher in the rural region, which can boost vaccination rates in Malaysia, especially among the poorest states with vast rural areas such as Kelantan and Sabah (61.4% and 62.9% total population with at least two doses of COVID-19 vaccine, respectively, compared to the national average of 79.1%) [3].

The identified potential barriers to implementing this extended pharmacy service in this study include the lack of pharmacists' training and maintaining patient safety, requiring better education and training for undergraduate and practising pharmacists. However, their positive attitude towards the factors necessary to implement this service, especially the certification in vaccine administration and continuous education and training workshops in vaccination, could overcome the potential barriers. Their willingness to spend on vaccine administration training could indicate their desire to cooperate in this service.

This study also found that pharmacists who have not received vaccination training have shown more barriers in providing the service than those who have. This study supported a prior study conducted in Poland in 2020, which found that pharmacists who had previously received vaccination training were more prepared and willing to administer vaccines. Untrained pharmacists expressed concerns about the workload, but reduced concerns after training. However, the trained pharmacists still felt that practical vaccination training alone was insufficient [4]. Since July 2021, Polish pharmacists have been qualified to administer vaccinations to healthy adults if they have completed the prerequisite theoretical and practical training and have obtained a certificate of vaccination competence [12].

Practical vaccination training may be a shorter course suited for busy practising pharmacists, while theoretical training can begin early during undergraduate study. A study among Hungarian pharmacists advocated for including pharmaceutical care, clinical therapeutics and clinical pharmacy competencies, including vaccination, in the curriculum to adapt to advances in pharmacy and give suitable preparation for pharmacists' expanding skill sets [13].

This study pointed out that pharmacies were not well-equipped to provide vaccination and the lack of space to administer and store vaccines as additional challenges in providing the service. Community pharmacists must be able to maintain the cold chain at the point of vaccine arrival, storage, handling and administration. Pharmaceutical-grade refrigerators (2 to 8 °C) and freezers (− 50 to − 15 °C) must be equipped with a temperature monitoring device and placed in a well-ventilated room with storage unit doors under standard indoor room temperatures. Facilities must have a stable electricity supply and equipped with an uninterruptible power supply (UPS) unit. Vaccines should be prepared in a designated area separate from any area where possibly contaminated goods are stored [14]. Vaccination can be done in the existing counselling room, which is mandatory for all community pharmacies in Malaysia, yet will have less room for other drug counselling sessions. The pharmacists also indicated that the supporting factors necessary to implement the vaccination services include relevant remuneration and reimbursement system, the possibility of providing a room to deliver vaccines and space to store vaccines. These findings are consistent with a study conducted in Canada [1], highlighting the need for reimbursement and the lack of space to administer vaccines as barriers to implementation.

This study discovered that pharmacists as staff were more willing to provide vaccination services to the public. Pharmacists as staff may have higher contact and rapport with patients. Similarly, a study among Egyptian pharmacists in 2020 shows that pharmacists as staff were more prepared for the COVID-19 pandemic and reported more suspected COVID-19 cases of their patients (*p* = 0.019) than pharmacists as managers [15]. However, in our study, pharmacists as staff perceived more barriers to providing such services due to the additional pharmacy service and the need to reimburse expenses and supplies of vaccines.

According to the pharmacists, conflict with other healthcare providers eligible to vaccinate could be a significant challenge. In the Philippines [16], Kuwait [17] and Egypt [18], physicians and other healthcare professionals' lack of support for clinical pharmacy practice was cited as the leading obstacle impeding its implementation. Currently, Malaysian CPs' issues of separating dispensing from prescribing still fail to be executed. In the current climate of conflict between community pharmacies and dispensing physicians, an addition of this extended pharmacy service is probably not well embraced by other healthcare professionals [19].

In Malaysia, pharmacists were not listed as the authorised person to administer poisons according to the Poison Act 1952, under Section 19(1), which only a registered medical practitioner or dentist can administer. Meanwhile, Section 20 of the Poison Act 1952 mentions that licensed pharmacists can only sell and supply poisons [20]. The findings of this study can support the suggestion of a prior study conducted in 2019 [19] that Malaysian healthcare policymakers and pharmaceutical professional bodies should review the roles of CPs in public vaccination programmes. We also hope that the findings of this study demonstrate the CPs' good intention to contribute to vaccination services, notwithstanding the challenges and issues that might be faced. The research findings can serve as a milestone for further education and training sessions for Malaysian CPs in the future, which could improve vaccination coverage. It could also be a starting point for Malaysian pharmacy schools by training pharmacists and undergraduate students by integrating vaccination education and practical sessions into their curriculum [19] to accomplish national vaccination goals.

The strength of this study includes using a validated English language questionnaire from previous research conducted in Saudi Arabia during the COVID-19 pandemic [4] and further validated to the Malaysian healthcare system in a similar period. In addition, the number of pharmacists who participated in this research exceeded the target sample size, indicating a strong willingness of Malaysian CPs to answer the questionnaire. The involvement of CPs in this COVID-19 pandemic, primarily in delivering the COVID-19 booster dose vaccines, can significantly increase the vaccination coverage rate in the Malaysian population.

The study limitations include a lack of participant coverage in the rural areas, particularly in East Malaysia. This is probably due to the reachability of the online questionnaire. A further study could target the CPs from rural areas by mailing the questionnaire together with a self-addressed stamped envelope (SASE). Future studies also could look into patient acceptance of vaccination by CPs. There is also a need to review the policies and practices of other countries that implement vaccine administration by CPs for the implementation in Malaysia.

## Conclusions

Malaysian community pharmacists were ready and willing to administer vaccination, although they expressed a need to improve their knowledge and had a high willingness to pay for vaccination training. Educational programmes and training workshops can prepare CPs for vaccination services and educate the public on vaccination. Besides training, operational guidance for providing vaccination services is necessary. These actions are crucial during the COVID-19 pandemic as pharmacists can significantly impact the vaccination coverage rate of patients and take an active part in combating infectious diseases.

## Data Availability

Not applicable.
